# Ceiling-mounted far-UVC fixtures reduce the surface bioburden in occupied clinical areas

**DOI:** 10.1017/ice.2025.62

**Published:** 2025-06

**Authors:** Emilie Hage Mogensen, Jacob Thyrsted Jensen, Søren Helbo Skaarup, Andreas Fløe Hvass, Cecilie Lynggaard Jeppesen, Maja Holst Rasmussen, Birgit Thorup Røge, Sara Moeslund Joensen, Stine Yde Nielsen, Elisabeth Bendstrup, Pernille Hauschildt, Anne Friesgaard Christensen, Christian Kanstrup Holm

**Affiliations:** 1 UV Medico A/S, Aarhus, Denmark; 2 Department of Biomedicine, Aarhus University, Aarhus, Denmark; 3 Department of Respiratory Diseases and Allergy, Aarhus University Hospital, Aarhus, Denmark; 4 Department of Clinical Medicine, Aarhus University, Aarhus, Denmark; 5 Department of Internal Medicine, Lillebaelt Hospital, Kolding, Denmark; 6 Department of Clinical Microbiology, Lillebaelt Hospital, Vejle, Denmark

## Abstract

Contaminated surfaces in clinics pose a pathogen transmission risk. Far ultraviolet-C light (UVC), with a favorable safety profile for human exposure, has the potential for continuous pathogen inactivation in occupied clinical areas. This study demonstrated real-world bioburden reduction on surfaces, despite frequent contamination from routine use by staff and patients in clinics.

## Introduction

Pathogens can persist on surfaces in clinics for prolonged periods, ranging from hours to months.^1^ Contaminated surfaces in clinics may contribute to pathogen transmission,^2^ highlighting the need for effective disinfection strategies that works in between cleaning sessions, where pathogens may accumulate and increase the risk of transmission.

Far-UVC (200–230 nm) has gained attention due to its reduced safety risks for human skin^3^ and eyes,^4^ when applied within regulatory limits, compared to conventional UVC light at 254 nm, which can cause significant harm to humans. This safety profile provides far-UVC with the potential to be used for pathogen inactivation in occupied clinical settings.

While the germicidal efficacy of far-UVC has been extensively demonstrated in benchtop studies,^5^ evidence from real-world applications in occupied environments with continuously contaminated surfaces remains limited. This study evaluated the efficacy of ceiling-mounted far-UVC fixtures in reducing bioburden on surfaces in two occupied clinical settings.

## Materials and methods

### Far-UVC device

The far-UVC device (UV222^TM^, UV Medico A/S, Denmark), equipped with a filtered krypton chloride excimer light source (Care 222®, Ushio Inc., Japan), emitted UV light at 222 nm within a 60° dispersion angle. The filter blocked emissions above 222 nm, and the irradiance was 13.7 µW/cm^2^ at one meter. Duty cycles were managed using UV222^TM^ (UV Medico A/S, Denmark) software.

### Far-UVC efficacy assessment in an outpatient waiting area

A respiratory disease outpatient waiting area in a Danish hospital was selected to evaluate far-UVC efficacy in an occupied clinical setting. Two ceiling-mounted far-UVC lamps were angled toward the plastic chairs. Using DiaLux EVO version 9.2 (DIAL GmbH, Germany) for UV exposure simulations, the average irradiance on chairs was determined to be 2 µW/cm^2^. The far-UVC lamps were set to a duty cycle that delivers approximately 400 µJ/cm² to the chair surfaces for each on-time period.

Bacterial sampling on the chairs was performed using Hygicult TPC dipslides (Adian, Finland) following the manufacturer’s protocol. Sampling was conducted in the afternoon over six days: three with far-UVC off and three with far-UVC on. Samples were collected from the same spots on the backrest and seat of each chair every time, yielding 72 samples. Colony-forming units (CFUs) were quantified after 24-hour incubation at 37°C.

### Far-UVC efficacy assessment on mobile workstations

Far-UVC efficacy was further evaluated in a medical ward of another Danish hospital, focusing on mobile workstations used by staff during rounds. A control ward with a similar layout, patient count, and workstation placement but without far-UVC was selected within the same hospital for comparison. Despite regular cleaning in accordance with hospital hygiene standards, intervals between sessions allowed pathogens to accumulate on the workstations.

Far-UVC lamps were mounted 132 cm above five workstations in the corridor, delivering 7.92 µW/cm² irradiance on average at the desk surface, and operating continuously for 18 hours daily (6 a.m.–12 a.m.). Weekly bacterial sampling was conducted over 14 weeks between 10 a.m. and 12 p.m. on the workstation desks in both wards, excluding any in use by medical staff during sampling. A total of 109 samples were collected using swab samplers with 1 mL Letheen Broth (3M, USA) following the manufacturer’s protocol, covering a 10×10 cm area on the workstation desks. Samples were split, with 200 µL transferred to a blood agar plate (bovine blood 5%, SSI Diagnostica, Denmark) and 800 µL to a Petrifilm (aerobic count, 3M, USA). CFUs were quantified on both the blood agar plates and Petrifilm after a 24-hour incubation at 37°C.

### Statistical analysis

Normal distributions were assessed using the Kolmogorov-Smirnov test. Paired *t*-tests were conducted to compare bioburden levels before and after far-UVC exposure in the outpatient waiting area, while unpaired Mann-Whitney *U*-tests compared bioburden on workstations between the far-UVC exposed and control wards. Statistical significance was set at 5%, and analyses were performed using GraphPad Prism version 10.2.0.

### Ethical concerns

No sensitive data were collected during the study, and informed consent was not required per local regulations. All interventions received approval from the chief physicians of the respective departments in both hospitals. The far-UVC device holds a CE mark, certifying compliance with European Union health and safety standards, and was operated within the International Commission on Non-Ionizing Radiation Protection’s (ICNIRP’s) threshold limit value of 23 mJ/cm².^6^

## Results

### Far-UVC reduces bioburden on surfaces in hospital outpatient waiting area despite continuous contamination from patients

**Figure 1.**
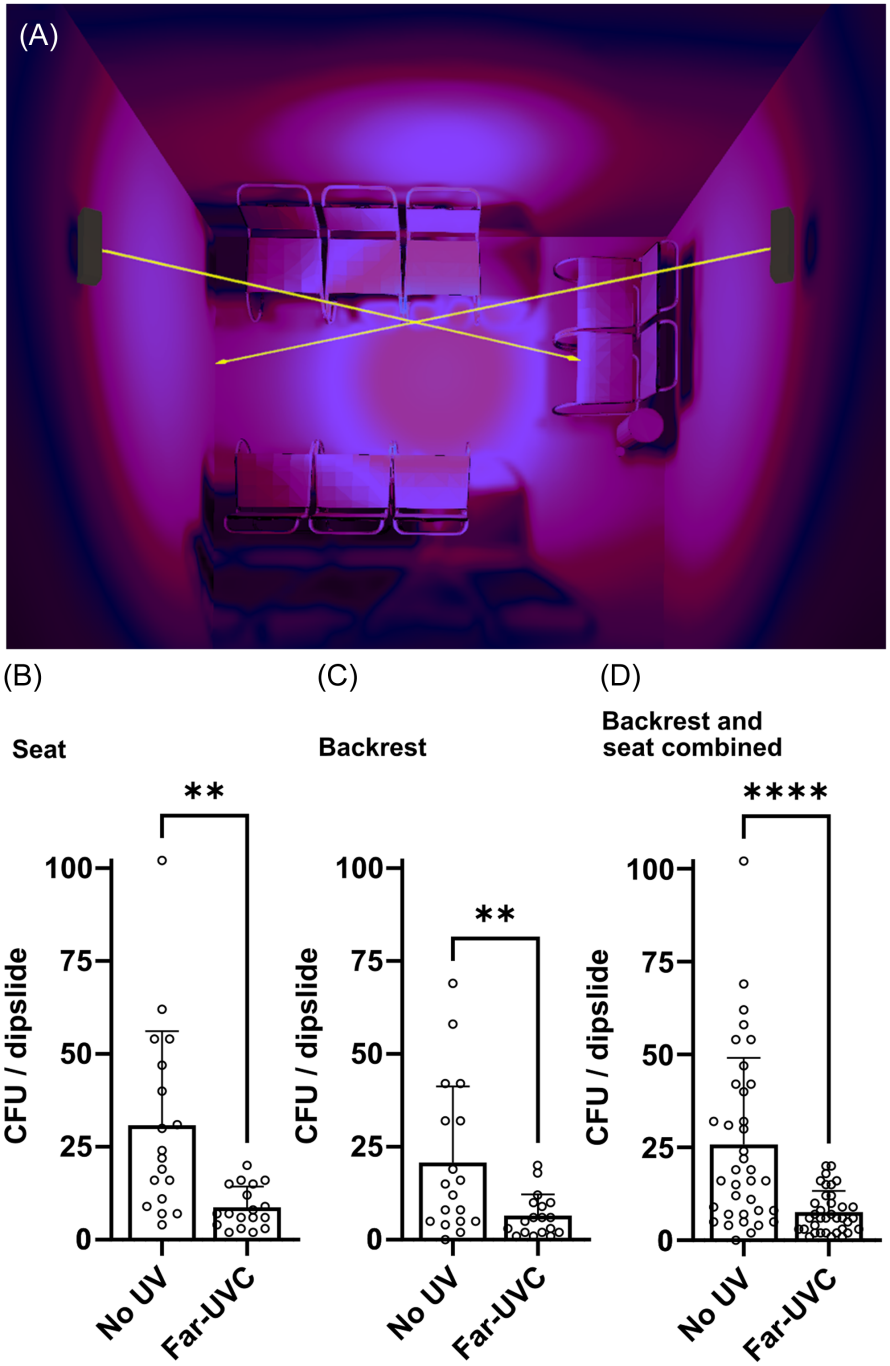
(A) Simulation of two ceiling-mounted far-UVC fixtures in an outpatient waiting area, with arrows indicating the light direction and surface exposure highlighted in magenta. CFUs per dipslide on seats (B), backrests (C), and combined (D) before and after far-UVC are shown. Mean values are represented as columns, standard deviations by vertical error bars, and each dot represents a single sample. Statistical evaluations were conducted using paired *t*-test (** *P* < .01, **** *P* < .0001).

The far-UVC exposure in the outpatient waiting area is depicted in Figure 1A. Far-UVC lamps significantly reduced mean CFU values on chair seats and backrests, from 30.8 to 8.7 and 20.7 to 6.5, respectively (Figure 1B and 1C). Combined, the mean CFU value decreased from 25.8 to 7.6, corresponding to a 70.54% reduction (Figure 1D, *P* < .0001, paired *t*-test). No far-UVC samples exceeded 20 CFUs, while control samples ranged from 0 to 102 CFUs, with 44.4% exceeding 20.

### Far-UVC reduces bioburden on medical workstations frequently used by staff

**Figure 2.**
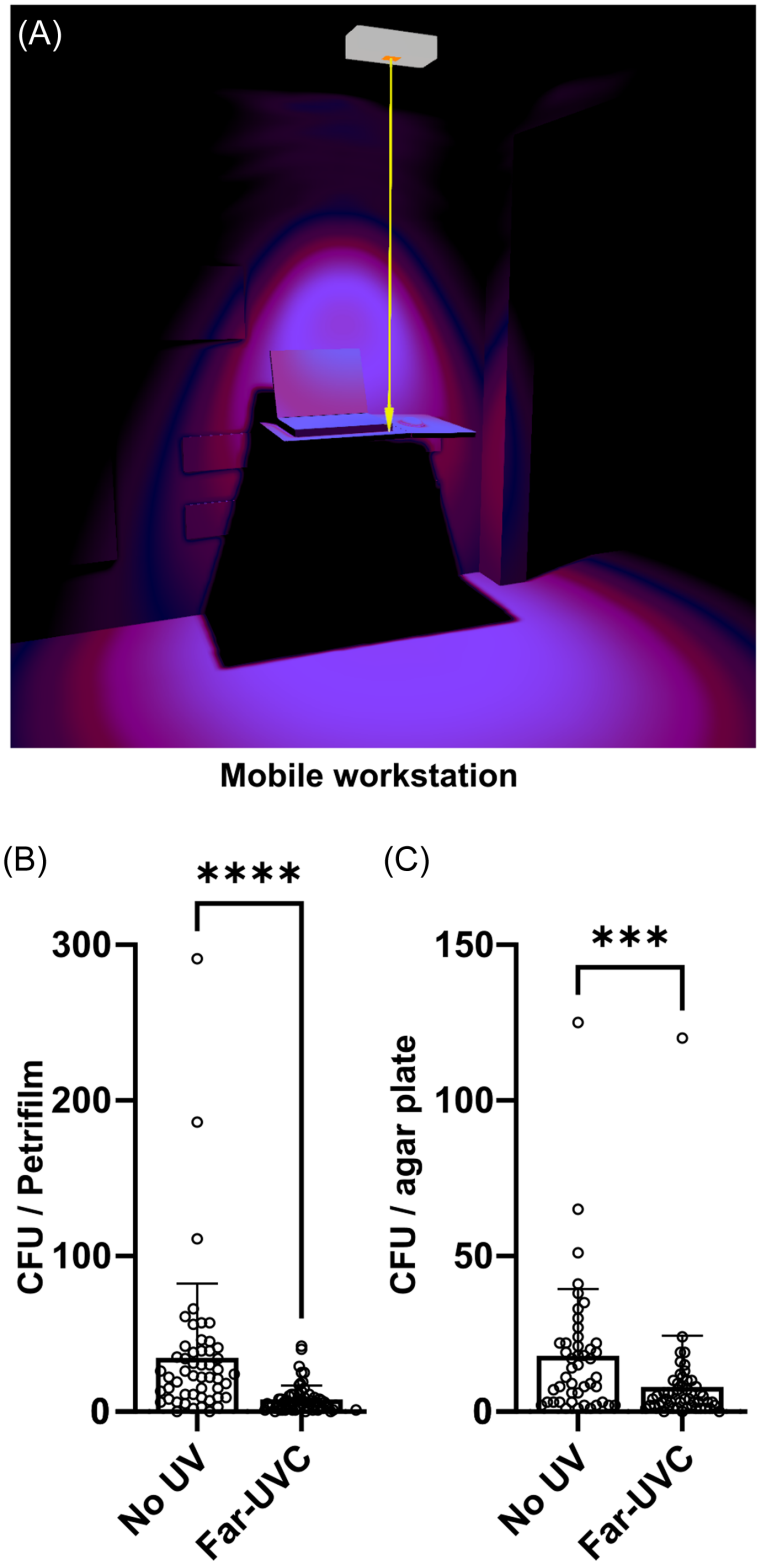
(A) Simulation of a ceiling-mounted far-UVC fixture above a medical workstation, with light direction indicated by an arrow and exposure highlighted in magenta. CFU counts quantified on both Petrifilm (B) and blood agar plates (C) are shown for far-UVC-irradiated workstations and non-irradiated workstations in a comparable ward. Columns represent mean CFU values, with dots indicating individual samples. Error bars represent standard deviations, and statistical significance is noted (Mann-Whitney *U*-test: *** *P* < .001, **** *P* < .0001).

The far-UVC exposure on workstations is depicted in Figure 2A. The presence and similarity of bioburden on the workstations in both wards, despite regular cleaning, were confirmed prior to the study (S1).

Mean CFU values from workstations in the non-irradiated control ward were 34.6 (Petrifilm) and 17.9 (blood agar plates) compared to 7.9 for both cultivation methods on the far-UVC exposed workstations (Figure 2B and 2C). This change represents percentage reductions of 77.2% (Petrifilm, Mann-Whitney *U*-test, *P* < .0001) and 55.9% (blood agar, Mann-Whitney *U*-test, *P* < .001).

## Discussion

We demonstrated that ceiling-mounted far-UVC fixtures reduced bioburden on surfaces in two occupied clinical settings. Contaminated surfaces in healthcare environments pose a potential risk of pathogen transmission,^2^ particularly as healthcare workers may be less likely to adhere to hygiene practices after contact with the patient surroundings compared to direct patient contact.^7^ While the presence of pathogens on surfaces does not necessarily lead to transmission, reducing bioburden likely lowers this risk.

The far-UVC fixtures were configured to operate autonomously, providing continuous disinfection without requiring staff intervention or additional training. This allowed patient care and existing procedures to remain unaffected while also minimizing the risk of user errors. However, far-UVC fixtures require regular maintenance, including routine inspections, to ensure long-term functionality, necessitating resource allocation. Another drawback is the initial cost of the devices, though this may be offset by their low daily operational requirements. As far-UVC does not physically remove contaminants from surfaces, it should be used as an addition to mechanical cleaning. This could potentially reduce levels of pathogens such as methicillin-resistant *Staphylococcus aureus*, vancomycin-resistant *Enterococci*, *Candida auris*, and *Clostridioides difficile,* as recently demonstrated in a study conducted in a hospital bathroom equipped with far-UVC fixtures.^8^ Studies suggest that combining far-UVC with manual cleaning offers synergistic benefits compared to manual cleaning alone.^9,10^

The study has limitations, including its focus on only two surfaces in two clinical settings and the lack of efficacy evaluation against different bacterial species, as well as viruses and fungi. Furthermore, the study also lacks an assessment of the broader and long-term impact of reducing bioburden in clinics. Therefore, comprehensive, large-scale studies are needed to determine whether far-UVC technology can significantly reduce hospital-acquired infections by continuously lowering the bioburden in clinical settings.

## Supporting information

Mogensen et al. supplementary material Mogensen et al. supplementary material

## Supplementary material

The supplementary material for this article can be found at https://doi.org/10.1017/ice.2025.62
