# Supplementary material for: Ceiling-mounted far-UVC fixtures reduce the surface bioburden in occupied clinical areas
Source: Infect Control Hosp Epidemiol. 2025 Apr 3;46(6):647–9. doi: 10.1017/ice.2025.62 (PMC12169949; doi:10.1017/ice.2025.62)
Supplement: Mogensen et al. supplementary material [file S0899823X25000625sup001.docx]

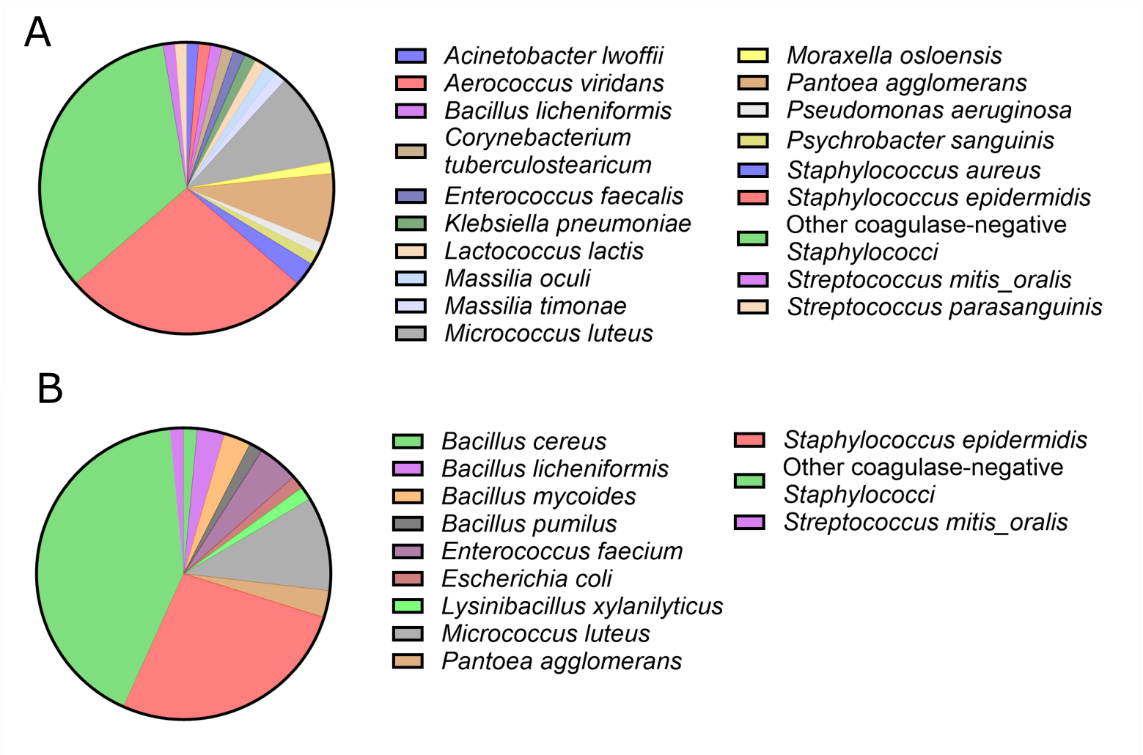


S1. Characterization of bacterial species on medical workstations in the (A) far-UVC and (B) control wards prior to activating the far-UVC fixtures. Bacterial species were identified using MALDI-TOF MS analysis. Among the identified species, some are associated with HAIs, including *Staphylococcus aureus aureus*, *Enterococcus faecalis*, *Klebsiella pneumonia*, *Pseudomonas aeruginosa*, and *Enterococcus faecium*. *Staphylococci* were the predominant species identified in both wards.
